# Innovative Approaches to Address Social Determinants of Health Among Adolescents and Young Adults

**DOI:** 10.1089/heq.2018.0011

**Published:** 2018-11-14

**Authors:** Kathleen P. Tebb, Gingi Pica, Lauren Twietmeyer, Angela Diaz, Claire D. Brindis

**Affiliations:** ^1^ Division of Adolescent and Young Adult Medicine, Department of Pediatrics, Benioff Children's Hospital and the Adolescent and Young Adult Health National Resource Center, University of California, San Francisco, California.; ^2^ ICF International, New York, New York.; ^3^ Icahn School of Medicine at Mount Sinai and Mount Sinai Adolescent Health Center, New York, New York.; ^4^ Philip R. Lee Institute for Health Policy Studies and the Adolescent and Young Adult Health National Resource Center, University of California, San Francisco, California.

**Keywords:** social determinants of health, interventions, adolescents, young adults

## Abstract

**Background:** Social determinants are the leading causes of health disparities. Yet health care systems have not systemically addressed social determinants of health as it pertains to adolescents and young adults (AYAs), among other populations in need. This study identified promising innovative programs across the United States.

**Methods:** Thirteen representatives from 10 programs completed a 45-min telephone interview. Transcripts were reviewed and analyzed to identify cross-cutting themes.

**Results:** Strategies included increasing access to quality, comprehensive and confidential health services, addressing the holistic needs of AYAs, collaborations across the health care delivery systems and other community services, and leveraging technology.

**Conclusion:** This study showcased innovative approaches to inform future efforts.

## Background

Disparities in health outcomes are a result of a myriad of socioecological factors that are linked to education, employment, income, discrimination based on race/ethnicity, gender, religion, sexual orientation, geographic location, mental health, and/or disability. These factors are commonly referred to as social determinants of health (SDOH).Worldwide, SDOH (e.g., structural factors such as national wealth, income inequality, and access to education) have the strongest impacts on adolescent health.^1^

In the health care system, providers traditionally respond to the presenting health issue, rather than working upstream to address the underlying factors (SDOH). Although most adolescents and young adult (AYA) health morbidity and mortality are largely preventable, providers face challenges in engaging with other systems that influence young people, such as schools, juvenile justice, and social service systems, to respond to the myriad needs of young people. The purpose of this study was to identify programs, across the United States, where the health care delivery system and the broader community are working together to address the root causes of health disparities (SDOH) and promote health equity for AYAs.

Despite been recent attention to SDOH, the concept is not new. In the United States, the concept of SDOH emerged in the early 1900s, but did not have a major influence on public policies until the 1950s.^1^ The first documented major disparities in health outcomes were not published until 1985^2^ and though this spurred additional initiatives,^3–5^ progress remained slow. In 2003, the Institute of Medicine reported disparities in health care access and quality among racial and ethnic minorities,^3^ and more recently, there has been greater attention to health disparities among AYAs, including non-whites, immigrants, lesbian, gay, bisexual, transgender, queer/questioning (LGBTQ), foster care, juvenile justice, homeless, and youth from underserved geographical areas.^6^ These populations are at a greater risk for poor health outcomes (e.g., injury, substance use, obesity, poor mental, sexual, oral, and other health problems)^7–13^ SDOH also contribute to health care access and utilization, which further impacts health outcomes.

While social determinants have been documented as contributing to negative outcomes, multisectoral approaches to implementing interventions aimed at eliminating or ameliorating the impact of social determinants is nascent, particularly in terms of partnerships with health care providers (who often are at the forefront of encountering the impact of poor living environments, food intake, trauma, etc.). The purpose of this study is to identify programs across the United States, working to address SDOH and identify strategies to inform how the health care system and the broader community can better work together in addressing SDOH and promote health equity.

## Methods

Programs were identified through a literature and Internet search using the terms “adolescent,” “young adult,” “teen,” “social determinants of health,” “health equity,” and “preventive care.” We also sought programs from known initiatives aimed at addressing SDOH: CDC's Racial and Ethnic Approaches to Community Health, National Prevention and Health Promotion Strategy, National Partnership for Action to End Health Disparities, Maternal and Child Health Bureau,^14–17^ and the Robert Wood Johnson Foundation.^18^ Fifteen programs were identified. We reached out to all 15 programs; 4 programs were no longer operating and 1 did not respond after four attempts. Programs came from a range of regions across the United States (California, Colorado, Massachusetts, New Mexico, New York, Rhode Island, and South Carolina). Table 1 provides a brief description of each program. We initially reached out to direct program leaders to interviews, and were referred to additional project staff to provide a more complete perspective on the program and/or topic. Thirteen individuals across 10 programs agreed to participate in 45-min semistructured telephone interviews that asked about their program, efforts to address SDOH, and challenges (Table 2). Interviews were recorded, transcribed, and analyzed to identify key themes. Transcripts were independently coded by two researchers and discrepancies were resolved through discussion. All participants provided consent to use their quotes and were allowed to review and approve them before publication. The University of California, San Francisco's IRB approved this study.

**Table 1.** Overview of Intervention Approaches by Region in the United States

Program	Funding	Brief description	AYA populations served
The Los Angeles Trust for Children's Health, Los Angeles, CA	CA-based Community Foundation & Endowment; CVS Caremark; Kaiser Permanente S. CA	Improve student achievement by increasing access to integrated health care and preventive services at 14 Wellness Centers	Adolescents at SBHCs, younger students and their families
One Degree, San Francisco, CA	Technology entrepreneurs, foundations, and government	A technology-driven organization linking low-income people with community resources	Low-income individuals, including AYAs and families
SHCIP, New Mexico and Colorado	Centers for Medicare and Medicaid	Identifies effective replicable strategies for enhancing health care quality through 22 SBHCs	School-age children and adolescents
The Door, New York, NY	Public/private; Title X federal funds; City & State Department of Health	Comprehensive health and development services to AYAs, including reproductive health, mental health, legal assistance, educational support, college preparation, and English tutoring	Youth ages 12–24 years
Housing Rx, Boston, MA	Boston Foundation's Health Starts at Home Initiative	Reduce housing instability among low-income families with young children	Low-income families with children
Progreso Latino, Rhode Island	CDC, grant funding, and fee-for-service	Connects Latinos and immigrants to free health care, dual-language adult education, and free/low-cost immigration legal services	Underserved and uninsured Latino and immigrant populations, including AYAs
Mount Sinai Adolescent Health Center, New York, NY	Government grants, foundations, clinic reimbursement, other gifts/donations	Delivers high-quality, comprehensive, confidential, and free health care. Outreach also provided through 24 middle- and high-school SBHCs	AYAs 10–24 yrs; low-income, uninsured, teen parents, and their children, immigrants, refuges, LGBTQ, transgender, homeless, and sex trafficked youth
New York City Teen Center, New York, NY	U.S. Department of Health and Human Services' Office of Adolescent Health; city tax levies	Connects youth with CBOs, schools, and clinics to promote evidence-based teen pregnancy prevention programs and access to sexual health care across three geographic communities in NYC	15,000 youth ages 15–19 years
Bronx Health REACH, Bronx, NY	CDC, National Center on Minority Health and Health Disparities, Johnson and Johnson, Johns Hopkins Community Healthcare Scholars	Reduce racial/ethnic disparities through health education and outreach, policy and system changes through evidence-based and community-informed interventions	Serves low-income youth and immigrant youth; almost all are Hispanic or African American
Spartanburg County Community Indicators Project South Carolina, Spartanburg, SC	CDC, Robert Wood Johnson Foundation, and Duke Endowment	Collect data on health indicators, set improvement goals, and work with CBOs to coordinate improvements	Residents of Spartanburg, South Carolina including children and AYAs

AYA, adolescents and young adult; LGBTQ, lesbian, gay, bisexual, transgender, queer/questioning; MSAHC, Mount Sinai Adolescent Health Center; NYCTC, New York City Teen Center; SCIP, Spartanburg Community Indicators Projects; SHCIP, School-Based Health Center Improvement Project.

**Table 2.** Questions for Semistructured Interviews

1. What are the key features of your program (or approach)—especially with regard to the intersection between health and community?
a. When did the program begin?
b. How did your approach to addressing some of the root causes of health disparities come about?
2. What do you think is most innovative about your approach?
3. Describe how you have worked across the health/community sectors
a. Which groups are you working with (e.g., juvenile justice, parks and recreation agencies, case management, health/clinic providers, transportation, other CBOs. etc)?
b. What was the motivation for each sector to come together?
4. Who does your program serve?
5. Does your program's activities specifically target adolescent and young adults? If yes, please explain and share any specific strategies you've used to tailor your approach to adolescents and/or young adults.
6. What is the size of your program?
a. If serve adolescents/young adults, how many do you serve/reach?
7. If you don't have adolescent specific strategies, how has working at the “family” level helped to assure that adolescents get services as well (intergenerational efforts).
8. What are the biggest challenges you have faced?
a. What were the barriers you encountered (if any) in bringing these sectors together (previous history of working together)?
9. What approaches have you used to address these challenges?
10. What are the sources of funding and other resources that you use to support your program?
11. What did this financial support contribute to the overall vision of what you/your agency are attempting to accomplish?
12. To what extent are these resources sustainable/how will you sustain and build on these efforts in the future?
13. Do you have any other comments you would like to add that I have not necessarily asked you about?

## Results

The primary focus of the interviews were to identify strategies in which health organizations worked in tandem with other types of community agencies to better respond to the myriad needs of their patients that went far beyond “clinic walls” to address SDOH. Participants were also asked to comment on challenges encountered. These findings are summarized hereunder. Table 3 provides quotes that further illustrate these themes.

**Table 3.** Illustrative Quotes to Accompany Key Themes in Addressing Social Determinants of Health

Approach to address SDOH	Interviewee quotes	Source of quote
3.1 Collaborating across multiple sectors of the community	“From our inception, we were a community coalition that recognized any one program in isolation would be insufficient to address disparities… We took a community-based participatory approach. We realized that to approach this work as a discrete project would not suffice if we were serious about addressing racial and ethnic health disparities.”	Charmaine Ruddock, Bronx Health
	“We noticed that our efforts to improve health outcomes in our county were very siloed and we were not sharing our efforts and lessons learned with each other. As a result, Spartanburg Community Indicators Project was started to share information, combine resources, and work in synergy.”	Dr. Kathleen Brady, University of South Carolina
	“We need to put the client at the center of our work. People don't need services from just one sector, their needs cut across a gamut of different services. We found that families utilize services from up to 12 non-profit organizations in order to get by.”	Rey Faustino, One Degree
3.2.a. Addressing root causes of poverty: education, job training, and career pathways	“We offer introductory emergency medical technician training and have partnered with a local culinary non-profit, who provides both meals to the youth, as well as culinary internship opportunities.”	Julie Shapiro, The Door
	“We work with STRIVE to help individuals acquire the skills and attitudes they need to overcome challenging circumstances, find sustained employment, and become valuable contributors to their families, employers and communities. For example we have STRIVE interns who spend 8 weeks working in the MSAHC research department learning research skills that are applicable across occupational fields.”	Michael Nembhard, MSAHC
3.2.b. Addressing root causes of poverty: Housing Rx	“Housing prescriptions is an incredibly adaptive model. Most communities already have the necessary resources to make this work for them.”	Dr. Megan Sandel, Housing Rx
3.2.c. Addressing root causes of poverty: teen pregnancy prevention	“We know for a fact that poverty drives teen pregnancy…. Teen pregnancy is not on a principal's mind, but they are accountable for graduation rates. If you approach a principal with data like ‘a person growing up in poverty is 3x more likely to get pregnant and drop out [of school]’—that's a compelling statement....and makes our program attractive to them.”	Estelle Raboni, NYCTC
	“To prevent social disparities, you have to deal with the root causes. For teen parents, especially those with a repeat teen birth, it is really hard to get out of poverty. With funding from the CDC and OAH were able to tackle this and reduce our teen birth rate.”	Polly Padgett, Mary Black Foundation
3.2.d. Addressing root causes of poverty: leveraging technology in health care to link to community supports	“We aim to reach the working poor, the 60–70% that are on the brink of poverty. They have Internet and the agency to look for resources… We are examining how to integrate it into a number of clinics through providers, health educators and when patients are discharged from the hospital so that people can find the resources that they need on One Degree.”	Rey Faustino, One Degree
3.3.a. Providing access to and delivery of holistic, comprehensive, and quality health care: delivery systems	“The Wellness Centers were conceived as a comprehensive, holistic, upstream approach to impact schools and neighborhoods…We were very intentional about addressing issues of equity and disparities in the system and needed a public health framework/population approach and policies to support this approach.”	Maryjane Puffer, The L.A. Trust
	“We provide a comprehensive holistic approach to working with young adults. Youth can access our services without any barriers, including the ability to pay, parental permission, and citizenship status. We also provide youth with a ‘warm hand off’ to a social worker to start an intervention. Most of our patients are hooked into services and will come back repeatedly to see social workers.”	Michael Nembhard, MSAHC
	“Ease of access is everything for adolescents. At The Door, they can come in for an internship, and then take a dance class and have a reproductive health visit.”	Julie Shapiro, The Door
3.3.b. Providing access to and delivery of holistic, comprehensive, and quality health care: data-driven approaches	“Funding from CHIPRA allowed us to make the Student Health Questionnaire electronic and generate alert reports. The electronic version (eSHQ) allowed us to better understand patient's needs and drive visit and follow-up care.”	McKane Sharff, SHCIP
	“We attribute our success to our data driven approach which is led by a broad- based community coalition.”	Polly Padgett, SCIP
	“We are very data heavy—it has given us a benchmark to track and assess the successes and identify areas for improvement in our community.”	Dr. Kathleen Brady, SCIP
3.4.a. Addressing special populations of AYAs to ameliorate historical injustice: staffing	“We are the only Latino-led social service organization in the state. We are founded by Latinos and over 90% of our staff is from our Latino community, many of us are first generation immigrants. We have a first-hand understanding of their needs. We provide a one-stop center and provide much needed support, especially for newly arrived immigrants.”	Mario Bueno, Progreso Latino
3.4.b. Addressing special populations of AYAs: to ameliorate historical injustice through safe and inclusive environments	“We create a supportive, nurturing and inclusive environment for all youth. Foster youth, LGBTQ, homeless youth want to be here because it is a fun and safe space for them.”	Julie Shapiro, The Door
	“We operate a transgender program that serves around 300 adolescents and young adults. We are one of the few agencies that provide hormone therapy for youth at age 18.”	Michael Nembhard, MSAHC
3.4.c. Addressing special populations of AYAs: engaging adolescents and young adults	“Youth engagement is at the heart of what we do. Youth are disproportionately affected by the broader society and are without an equitable voice in determining their own futures. Students help keep adult institutions grounded, relevant and effective.”	Maryjane Puffer, The L.A. Trust
	“We incorporate youth in our work. For example, peer health educators helped develop our website and make it more youth-friendly.”	Moya Brown, MSAHC
	“We utilized youth engagement as a tool to drive traffic to the SBHCs. One strategy included being visible at lunch and tabling around a specific health topic.”	McKane Sharff, SHCIP
3.5. Advocating for public policies to address SDOH	“We knew statistically teen moms were not coming back for contraceptives at post-partum visits. We worked on changing this policy to allow IUDs to be inserted post-partum, at delivery. This initiative had a huge impact—a decrease in repeat births from 38% to 22%.”	Polly Padgett SCIP
	“We were very intentional about addressing issues of equity and disparities in the system and needed a public health framework/population approach and policies to support this approach.”	Maryjane Puffer, The L.A. Trust
3.6. Need for flexible and diverse funding sources	“There is money in Accountable Care Organizations from the ACA, to fund community services like our housing prescription program. This funding is flexible and can be used to address issues like housing and food insecurities. This funding is now in jeopardy if the ACA is blocked.”	Megan Sandel, Housing Prescriptions for Health
	“The CDC decided that unlike their past efforts where they funded a particular agency, program or academic institution, they were going to directly fund communities. From the beginning, they did not have a prescriptive approach. This allowed us to give sub- awards. It allowed communities to have autonomy and come to the table as an equal partner”	Charmaine Ruddock, Bronx Health REACH
	“Our program has been sustainable in large part because of the diversity of our funding streams. If we take a hit in one area, we grow in another. We blend and leverage all of our funding sources but it is all seamless from the perspective of our youth.”	Julie Shapiro, The Door

SDOH, social determinants of health.

All interviewees stated that it was critical to form community partnerships across multiple sectors to address SDOH. Most individuals need services that span different programs, for example, health, education, juvenile justice, and social services. Benefits of these partnerships include raising awareness of community programs, increasing AYAs' access to services, identifying gaps, and building support for and capacity to meet the needs of AYAs (“3.1” in Table 3).

### Addressing poverty, a key SDOH

Interviewees universally acknowledged poverty as an important SDOH. Programs implemented a number of different strategies to address this SDOH. The most common approach involved educational supports, job training, and career pathways. For instance, the Door's embeds health care services for AYAs within a larger positive youth development program that offers comprehensive career and education programs and opportunities to develop job and life skills. In addition, the Mount Sinai Adolescent Health Center (MSAHC) provides comprehensive integrated health services, leadership training, education and skill development, and legal and social services to help AYAs overcome challenging circumstances such as the ability to return to or stay in school, and find sustained employment (“3.2” in Table 3). Similarly, the The Los Angeles Trust for Children's Health (L.A. Trust) supports comprehensive access to health and wellness and also provides AYAs from low-income, high-risk neighborhoods with skill development, and employment opportunities in health care through employer, community, and state college partnerships.

Prior research shows that poverty is linked with housing instability that, in turn, places youth and their families at risk of adverse health outcomes.^19^ Boston Medical Center launched Housing Prescriptions (Rx) to mitigate adverse health outcomes that stem from housing instability. In this approach, the medical setting identifies at-risk families and provides them with a housing Rx that is used to link participants with a community-based case manager that provides specialized housing support, linkages to resources, and fast-tracks eligible families into public housing units (“3.3” in Table 3).

Poverty is also a predictive factor in teen pregnancy rates, which, in turn, increases the risk of poverty and poor health outcomes of the teen parent(s) and their offspring. A couple of programs focused on addressing this SDOH through teen pregnancy prevention efforts that include evidence-based, comprehensive and confidential, sexual health education and services. For example, the Spartanburg Community Indicators Projects (SCIP) worked with Medicaid to change state-level policies to allow intrauterine device placement at delivery and post-partum visits to reduce repeat teen pregnancies (“3.4” in Table 3). In another example, the L.A. Trust partnered with researchers to develop and evaluate a mobile health application to provide patient-centered evidence-based comprehensive contraceptive information and access to reduce disparities in unintended pregnancies among Latina adolescents.^20^ Other programs such as the Door and MSACH also incorporate teen pregnancy prevention efforts that are multifaceted and include educational, medical, and social/economic approaches.

In yet another approach, One Degree, a nonprofit technology-driven organization, strives to “empower people to create a path out of poverty for themselves and their communities.” Through their web and mobile platform (1degree.com), low-income individuals and families can get linked with community resources (e.g., housing, health care, and food banks). This tool has been integrated in a number of health clinics to address food insecurities and other needs associated with poverty. A growing number of pediatric providers are screening families for food insecurities as part of the American Academy of Pediatrics efforts to address this SDOH.^21^ When a family is identified as experiencing food insecurities, the One Degree application (app) can be used to link families to local resources (complete with walking directions and public transportation routes).

### Providing holistic and comprehensive health care with linkages to other services

Many programs identified unequal access to quality health care services as a major SDOH and focused efforts on promoting equity in AYAs' access to health care and improving health care quality for all AYAs. In particular, MSAHC serves AYAs regardless of their ability to pay or their insurance status and provides holistic, confidential, comprehensive, integrated medical, sexual and reproductive health, dental, optical, behavioral and mental health, prevention, and support services. The Door also provides a wide range of services, in one location, that are free and confidential, including reproductive health care and education, mental health counseling and crisis assistance, legal assistance, academic support, job training and placement, supportive housing, recreational and arts activities, and nutritious meals. The New York City Teen Center (NYCTC) brought together youth, community-based organizations, schools, citywide agencies, and >66 teen-friendly clinics to ensure that every teen has access to high-quality comprehensive health services. The L.A. Trust supports a network of Wellness Centers that serves students in Los Angeles and provides comprehensive care, including oral health, asthma, reproductive health, substance abuse prevention, and mental health services (“3.3.a.” in Table 3). They also utilize promotoras^*^ who engage with families to support communication about healthy relationships and behaviors.

A few programs also utilize data-driven approaches to identify needs, monitor progress, and inform continuous quality improvement efforts. For example, the School-Based Health Center Improvement Project (SHCIP) enhancing the quality of health care for youth with a special focus on increasing access for adolescents, with the lowest rates of primary care use. They implemented an electronic Student Health Questionnaire to assess for risk and protective factors through an iPad at the medical appointment. Providers could view the results immediately and use them to guide the visit. This approach ensures all youth receive comprehensive screening and appropriate care. MSAHC also uses technology to promote quality of care through Health Squad that encourages AYAs to ask questions and request support 24/7 for a response within one business day. It also provides AYAs with medication reminders and health information. Using data-driven quality improvement approaches, for example, SCIP collects county-level population data on health outcomes and trends to collectively decide on focus areas, set improvement goals, coordinate improvement efforts, and track and report on progress (“3.3.b,” in Table 3).

In addition, there was wide-spread recognition that special populations of AYAs needed targeted approaches to ensure access as long-standing discrimination based on race/ethnicity, gender, religion, sexual orientation, geographic location, mental health, disability, age, income, etc that has limited health care access. Approaches to mitigate this SDOH include hiring staff who reflect the background of the populations they serve (“3.4.a.” in Table 3) and creating safe/welcoming environments for all AYAs (“3.4.b.” in Table 3). Engaging youth was central to several programs to ensure youth-centered and inclusive programming. For example, MSAHC utilized peer educators to develop their teen-friendly website, and the L.A. Trust engaged youth advisors to inform the organization and board of directors about students' perceptions of the Wellness Centers to improve student utilization (“3.4.c.” in Table 3).

### Advocating for broader public policies to address SDOH

Interviewees revealed that policy changes are a critical component to promoting social justice and health equity. For example, Bronx Health REACH created policies to address SDOH at the local and state level.^22^ They instituted school-based initiatives, including a policy to replace whole milk in all 1579 public schools and legislation requiring students to receive state-mandated physical education.^23^ They launched #Not62 campaign for a healthy Bronx, which incorporates a community call to action for elected officials, faith-based leaders, health care executives, and community members to create the infrastructure needed to address social and economic factors to promote health equity^24^ (“3.5” in Table 3).

### Challenges

The most significant challenge all interviewees faced was a lack of stable funding to sustain intervention efforts. Funding was often short term and/or targeted. Several interviewees from organizations, including the Door, Progresso Latino, the L.A. Trust, NYCTC, MSAHC, and Bronx Health REACH, stated that diverse funding was critical to their sustainability as it enabled them to withstand cuts to any one particular funding source. In addition, Housing Rx utilizes community partners, representing different funding streams, to maximize the benefits an individual may be eligible to receive (“3.6” in Table 3). However, this approach created challenges in managing different funders' requirements that targeted different problem areas or populations with different eligibility criteria, different reporting requirements, etc. It also makes it more difficult to provide seamless services. Others mentioned challenges with infrastructure and capacity to meet the needs of AYAs, especially in under-resourced settings. A few commented on challenges integrating new technologies citing a number of issues including staff training, availability of IT support for wireless connections, and application updates. Some also mentioned that it can be overwhelming for community agencies and providers to respond to SDOH as there are many complexities to any given SDOH. A couple of participants also noted a lack of political will for broader solutions to address root causes of health disparities.

## Conclusion

Addressing SDOH for AYAs is complex, yet this study provides examples of existing efforts across the United States that can offer some guidance for future endeavors. All of the programs featured in this study created linkages between the health sector with other influential community stakeholders to improve coordination across multiple sectors of health, social, and community-based programs and services. This coordinated approach was evident across each of the strategies aimed at addressing SDOH. For instance, in tackling poverty, the health and social service sectors worked together in implementing a range of approaches (job/skill preparation, housing prescriptions, and teen pregnancy prevention effort). Inequalities in access to health care and quality of health services were another major SDOH many programs tackled through providing holistic comprehensive care that was data driven, safe, inclusive, and engaged AYAs to ensure programming is relevant and effective at meeting their unique needs. In addition, a few programs also targeted the “upstream” social-ecological factors through advocating for broader policies to address root causes of poverty, unstable housing, food insecurity, and other factors.

Innovative and stable financing strategies are needed to address SDOH, outside of the hospital/clinic setting, to promote access to healthy foods, housing, transportation, employment, etc. as the links between these factors and health outcomes are well established. There is also a need for more comprehensive and well-evaluated approaches to tackle these complex and difficult challenges, including the root causes of discrimination and poverty. A social disparity and an equity lens need to underlie the strategies adopted by a wide array of health and nonhealth providers, programs and institutions that interact directly and indirectly with AYAs and their families.

## Footnotes

^*^ Promotoras are Hispanic/Latina women in the community who are trained to provide health information to other members of their community.

## Abbreviations Used

AYA: adolescents and young adult
LGBTQ: lesbian, gay, bisexual, transgender, queer/questioning
MSAHC: Mount Sinai Adolescent Health Center
NYCTC: New York City Teen Center
SCIP: Spartanburg Community Indicators Projects
SDOH: social determinants of health
SHCIP: School-Based Health Center Improvement Project
